# Health Indicators of US Older Adults Who Received or Did Not Receive Meals Funded by the Older Americans Act

**DOI:** 10.1155/2017/2160819

**Published:** 2017-10-22

**Authors:** Edgar R. Vieira, Joan A. Vaccaro, Gustavo G. Zarini, Fatma G. Huffman

**Affiliations:** ^1^Department of Physical Therapy, Florida International University, MMC, 11200 SW 8th St., AHC3-430, Miami, FL 33199, USA; ^2^Department of Dietetics and Nutrition, Florida International University, MMC, 11200 SW 8th St., AHC5-324, Miami, FL 33199, USA; ^3^Department of Dietetics and Nutrition, Florida International University, MMC, 11200 SW 8th St., AHC5-300, Miami, FL 33199, USA; ^4^Department of Dietetics and Nutrition, Florida International University, 11200 SW 8th St., AHC-5, Room 326, Miami, FL 33199, USA

## Abstract

**Background:**

The Older Americans Act (OAA) requires that nutrition programs provide meals and related nutrition services that promote health and help manage chronic diseases. The purpose of this study was to compare health status, food security, functional limitations, and chronic diseases of older adults who received or did not receive OAA meals using data from a representative sample of US adults.

**Methods:**

Data were from the National Health and Nutrition Surveys 2011–2014 for 2,392 older adults ≥ 65 years of age, including 187 Mexican Americans, 212 other Hispanics, 521 non-Hispanic Blacks, 219 non-Hispanic Asians, and 1253 non-Hispanic Whites.

**Results:**

Those receiving OAA meals had higher percent of food insecurity and functional disabilities. Adjusting for potential confounders, adults who received OAA meals had higher odds of emphysema (OR = 2.02; 1.05, 3.89) and lower odds of good-to-excellent health (OR = 0.52; 0.36, 0.77). Women and minorities had poorer health status compared to non-Hispanic Whites.

**Conclusion:**

A higher proportion of older adults who received nutritional services reported poorer health as compared to older adults who do not participate in these services. Future studies should assess nutritional adequacy for older adults who participate in nutritional programs comparing sex and race/ethnicity.

## 1. Introduction

Nutrition is the cornerstone of healthy aging, yet millions of older adults lack access to sufficient quantities of quality food, placing them at risk for disability, high cholesterol, high blood pressure, obesity, heart disease, cancer, and diabetes [1]. Good nutrition is vital for older adults to maintain health and independence, and it is especially compromised among those in social and economic need [1]. Food insecurity is related to medication nonadherence in low-income, older adults [2]. Medication nonadherence results in chronic disease mismanagement and increased morbidity and mortality [3].

The Older Americans Act (OAA) requires that nutrition programs provide meals and related nutrition services that promote health and help manage chronic diseases [4]. The OAA is now part of the mandate of the Administration for Community Living (ACL) within the Department of Health and Human Services (DHHS). The nutrition program is for adults 60 years of age and older; however, it concentrates assistance on persons with the greatest social or economic need, such as low-income older adults living in rural areas [5]. The ACL provides both home-delivered and congregate meals as part of the agency's fulfillment of the OAA [6]. The states have control for providing the programs and can provide one, two, or three meals 5 to 7 days per week [6]. Each meal must provide one-third of the recommended dietary allowances (RDAs). Congregate meals and government home-delivered meals are critical services provided to help older adults remain independent [6].

According to the Academy of Nutrition and Dietetics, there is limited research on the health outcomes of older adults who participate in the OAA nutrition programs [7]. Older adults receiving congregate or home-delivered meals from the OAA were found to have greater functional decline and more illnesses and to be poorer and at greater risk of institutionalization than the general older adult population [8]. Adults 65 years of age and older are the fastest growing segment of the population, increasing from 36.6 million in 2005 to 47.8 million in 2015 (a 30% increase); their segment is estimated to double by 2060 [9]. Thirty percent of community-dwelling adults receiving Medicare (aged ≥ 65 years) reported difficulty in performing one or more ADLs in 2013 [9]. Limitations in active daily living skills are projected to increase from 2.7 million (2014) to 12.2 million in 2035 among community-dwelling adults 65 years of age and older [10]. Due to the importance of nutrition in healthy aging, the purpose of this study was to compare health status, food security, functional limitations, and chronic diseases of older adults who received or did not receive OAA meals using data from a representative sample of US older adults.

## 2. Methods

### 2.1. Description of Sample

We evaluated data from 19,151 people who completed the National Health and Nutrition Examination Survey (NHANES) between 2011 and 2014 that is available for public use [11]. The inclusion criteria were being 65 years of age or older, self-identifying as Mexican American (MA), other Hispanic (OH), non-Hispanic Black (NHB), non-Hispanic Asian (NHA), or non-Hispanic White (NHW), and responding to the question on receiving or not OAA meals (congregate meals or government home-delivered meals). Age ≥ 65 years was a criterion because health insurance is then available through Medicare, even though nutrition programs are already available starting at 60 years old. Those who self-identified as “other” for race/ethnicity which included mixed races were not included due to the small sample size. Based on the inclusion criteria, our final sample size was 2,392 older adults, including 187 MA, 212 OH, 521 NHB, 219 NHA, and 1253 NHW.

### 2.2. Source of Data and Compliance with Ethical Standards

The NHANES applies a complex, stratified, multistage probability cluster sampling design to obtain a nationally representative sample of the US civilian, noninstitutionalized population. These surveys contain data for approximately 20,000 individuals (5000 per year) of all ages and were generated under the auspices of the National Center for Health Statistics (NCHS), a division of the Centers for Disease Control and Prevention (CDC), a part of the US DHHS. All participants read, understood, and signed informed consent forms. Separate informed consent forms were signed by participants depending on whether they participated in the interview and health examination or just the interview. For this study, the mobile examination center (MEC) sample weight was used to account for unequal probabilities of selection and nonresponse and to conform to the population distribution. Weight and height were measured in a mobile examination center using standardized techniques and equipment. Body mass index (BMI) was calculated as weight in kilograms divided by height in meters squared (kg/m^2^). Detailed information concerning the NHANES data collection procedures is available at the NCHS website: https://www.cdc.gov/nchs/ [11].

### 2.3. Demographic Variables

*Body mass index (BMI) categories* corresponded to National Institutes of Health (NIH) classification [12]. However, there were not enough individuals in the underweight category (less than 2%), so the underweight category was combined with normal weight category. The BMI categories were as follows: underweight to normal weight (BMI < 25 kg/m^2^), overweight (BMI ≥ 25 and <30 kg/m^2^), Obesity I (BMI ≥ 30 and <35 kg/m^2^), Obesity II (BMI ≥ 35 and <40 kg/m^2^), and Obesity III (BMI ≥ 40 kg/m^2^).

*Food security level* was classified based on household interview data using a validated questionnaire, US Food Security Survey Module, developed by the United States Department of Agriculture (USDA) [13]. People were classified as having full food security level, marginal food security level, low food security level, or very low food security level. In addition, food security was assessed based on being afraid of running out of food over the past 12 months (yes/no).* Poverty level* was measured as the ratio of monthly income to poverty, based on the 2011–2014 Department of Health and Human Services (HHS) poverty guidelines, and used by NHANES to calculate the index.* Poverty level* was constructed as a binary variable with a ratio < 1 considered as poverty (as per the US government).

*Marital status* was classified as “currently married/partnered or not” (single, widowed, or divorced). Finally,* smoking status* was classified as “current smoking or not” (including former smokers and never smokers).

### 2.4. Health Status

*Physical function* was classified as impaired when people said they had limitations for the amount of work they could do, or that they needed to use walking equipment (assistive devices). The* presence of a disease* was assessed based on an affirmative response to the question, “Have you ever been told by a doctor or other medical professional that you had _____________ (the disease)?”* Cognitive status* was classified as impaired when people said they had problems with memory.* The actual question asked to the individuals was as follows*: “Are you limited in any way because of difficulty remembering or because you experience periodic confusion?” And the response was dichotomous (yes/no).* Self-rated health* was assessed using the question, “In the past 12 months, how would you rate your health?” The original five categories (excellent, very good, good, fair, and poor) were collapsed to a binary variable: poor-to-fair or good-to-excellent health. Self-rated health has been validated against actual health in older adults, and it is an independent predictor of mortality [14–16].

### 2.5. Data Analysis

Descriptive statistics of the sample characteristics were presented as cross-tabulations (frequencies in percent and 95 percent confidence intervals) comparing those who received and did not receive OAA funded meals (congregate meals and/or home-delivered meals). Significance was based on the adjusted *F*, a variant of the second-order Rao-Scott adjusted chi-square statistic and its degrees of freedom. A logistic regression model was constructed using major demographics (gender, race/ethnicity, and marital status) and health indicators that were significant in the cross-tabulation analysis. All data were analyzed with the module for complex sample design analysis and sample weights, considering the differential probabilities of selection, with the Statistical Package for the Social Sciences (IBM SPSS, version 24) using the Taylor series linearization. For logistic regression, the Wald *F* test statistic was used for model fit with a model effect of *p* < .01 and a correct classification of cases ≥ 75%. To achieve model fit, all potential adjustment variables were added, retained only with a partial *p* value of .2 or less. A *p* value of less than .05 (two-sided) was considered statistically significant for variables.

## 3. Results

Table 1 presents the demographics and health indicators of the sample. Approximately 10% of older adults reported having received congregate meals and/or government home-delivered meals. There was a slightly larger proportion of females in the sample; most participants were non-Hispanic Whites and married. Less than 10% were smokers, but more than two-thirds were overweight or obese. About 10% reported fear of running out of food. More than three-fourths of the sample considered their health to be good to excellent, and the percentage of stroke, emphysema, and memory problems varied between 5 and 15%. About one-fifth of the population required an assistive device for walking and about 30% reported limitations in the amount of work they could do.

The frequency of those receiving OAA meals (congregate or home-delivered) was 287 (unweighted count, weighted frequency: 10.1% (8.0, 12.7)) as compared to those not receiving these services (2105, unweighted count, weighted frequency: 89.9% (87.3, 92.0)). Differences between participants who received and did not receive meals funded by the OAA are shown in Table 2. Twice the number of females, as compared to males, participate in the nutrition programs (home-delivered meals, congregate meals, or both). Those who reported using nutritional services had a higher percent of reporting being single as compared to married/partnered individuals, having had a stroke, having memory problems, being food insecure, having emphysema, needing equipment for walking, being limited in the work they could do, and having poorer health.

Reports of chronic diseases were compared for program participants versus nonparticipants using complex cross-tabulation. Having any cardiovascular disease, stroke, coronary heart disease, and emphysema was significantly more prevalent (higher percent) for individuals receiving meals as compared to their counterparts. Diabetes, high cholesterol, high blood pressure, arthritis, bronchitis, and cancer were not significantly different. A higher percentage of persons receiving OAA meals (congregate or meals on wheels) were living at or below the poverty level compared to those not receiving meals. A final logistic regression model is presented by Table 3. Participants did not differ by gender or race/ethnicity, but married or partnered ones had lower odds of participation as compared to single, widowed, or divorced individuals. Individuals reporting going to congregate meals or receiving government meals had poorer self-rated health (SRH) and greater odds of emphysema. Food insecurity and physical function (needing equipment to walk and limited in the amount of work one can perform) were no longer significant, adjusting for demographics. The model was fit with *p* < .001 and 90.1% correct classification (Table 3).

Cross-tabulations of health factors were performed for the combined population (participants and nonparticipants of OAA) by gender and race/ethnicity. Females had a higher percent reporting memory problems [14.8 (23.8, 17.0) versus 10.6 (9.1, 12.3)], *p* = .001, food insecurity (fear of running out of food) [11.6 (9.9, 13.6) versus 8.5 (6.9, 10.4)], *p* = .005, and needing walking equipment [21.7 (19.0, 24.7) versus 17.0 (13.9, 20.5)] as compared to men, *p* = .027. Mexican Americans and other Hispanics had a higher percent of reporting memory problems [23.5 (17.2, 31.2) and 24.7 (18.7, 31.9)], respectively, as compared to NHW [11.7 (9.9, 13.8)], *p* < .001. Other Hispanics [27.5 (22.5, 33.2)] and NHB [28.3 (23.4, 32.5)] had a higher percent of reported needing walking equipment as compared to NHW [18.2 (15.6, 21.3)], *p* < .001. Other Hispanics had a higher percent of reporting having to limit the amount of work they can do as compared to NHW [40.4 (34.2, 46.9) versus 30.5 (26.5, 34.8)], *p* = .010. Higher percent of food insecurity (fear of running out of food) was reported for MA [28.5 (22.3, 35.6)], OH [30.7 (22.9, 39.7)], and NHB [22.0 (16.4, 28.9)] as compared to NHW [7.2 (6.0, 8.6)], *p* < .001.

## 4. Discussion

We found a higher proportion of older adults (unadjusted) who receive nutritional services (home-delivered meals, congregate meals, or both) to have physical disabilities, report poorer health, and suffer from debilitating chronic diseases as compared to older adults who do not participate in these services. Even after adjusting for potential confounders, poorer self-rated health and emphysema were more likely for those receiving nutrition services. Our results parallel data from a national survey of OAA participants where 71% of home-delivered meal participants and 57% of congregate meal participants had five or more chronic diseases [8]; however, this survey did not compare OAA participants with nonparticipants. The Administration for Community Living [6] recommends that congregate meal sites have fitness classes to improve the health and function of participants. Our study did not have data on exercise class attendance; however, there is some evidence to support fitness classes; physical activity classes at congregate meal sites improved physical activity effort and physical function for older adults, even those with a history of depression [17, 18].

Food insecurity was a contributing factor of poor physical function for congregate meal attendees [19]. Older adults attending congregate meals have a higher percent of food insecurity (18.1%) [20] compared to the national average in older adults (6.5%) [21]. The goal of OAA is to reach older adults with food insecurity, yet there are instances of inequity among some communities. Weddle and colleagues [22] reported that, for community-dwelling, older African Americans, nutrition risk was higher among nonparticipants compared to participants of congregate meal services. Home-delivered meals improved dietary status and reduced food insecurity of recipients in 6 out of 8 studies, according to a systematic review [23]. Meal enhancements such as delivered snacks and local fruits and vegetables and nutritional supplements for those that need them have demonstrated reductions in food insecurity and improvements in functional status [24]. Nutritional adequacy may not be equal across demographics. Women and Blacks who received home-delivered meals had lower nutritional intake compared to their counterparts, regardless of health condition [25]. Food adequacy may be a confounder for health status (chronic disease and physical function) and program participation. Participants receiving OAA meals had a higher percent of food insecurity as defined by “fear of running out of food” as compared to those not receiving OAA meals. Although participation in nutritional services (home-delivered meals, congregate meals, or both) did not differ by race/ethnicity in the present study, there were a greater proportion of minorities with food insecurity as compared to NHW [26]. Older Blacks and Hispanics have more than twice the rate of food insecurity as compared to NHW [26]. A greater proportion of restricted eating was reported for Blacks who had food insecurity and who were obese as compared to their White counterparts [27]. In an earlier study, investigators reported that a higher percentage of older adults with food insecurity were obese (37%) as compared to older adults, nationally (23%) [28].

There was a significant percent of older adults with obesity in our study. Malnutrition is possible for older adults with obesity when diet quality is inadequate [29]. We found no significant difference in obesity for participants versus nonparticipants. This may be due to the alarmingly high rates of obesity among older adults, particularly women. Obesity was associated with abnormal eating behaviors, certain food group intake, and mental health symptoms in a predominately African American and female, urban congregate meal site [30]. Obesity rates from 2011–2014 were reported as 41% (those aged 65–74 years) and 31% (those aged 75 years and older) for women and 24% (those aged 65–74 years) and 13% (those aged 75 years and older) for men [31]. There are controversial reports for weight-loss interventions with physical activity as a treatment for physical disability in older adults [32]. Obesity was associated with disabilities for older adults (17.4%) [19]. Furthermore, there may be differences by sex and race/ethnicity for overweight and mild obesity protective effects with exercise [32]. Prior to developing nutrition interventions at community sites for older adults with obesity, more evidence is needed on how to treat this population [24].

Emphysema and poorer self-rated health were more prevalent for those receiving OAA meals as compared to their counterparts. The current study supports previous findings that older adults participating in nutrition programs have poorer health and lower socioeconomic status, compared to the general population of older adults [8]; however, functional decline did not differ by participation level, adjusting for sociodemographics. The current study indicated that participation/nonparticipation in nutrition programs did not differ by race/ethnicity and that women and minorities had poorer health status than non-Hispanic Whites, regardless of their participation level in nutrition programs. These findings suggest that greater outreach is needed for OAA meals and that interventions to improve health status should be addressed within these programs to help reduce health disparities.

This study has several limitations. Although data was from a nationally representative sample of US older adults, the study was cross-sectional and cause and effect cannot be assumed. The percent of home-delivered meal participants in our study was less than 10%, so they were combined with congregate meal participants for all analyses. Although the interviewers were trained to administer questions, some responses by participants might have been what they perceived as socially acceptable. Memory loss was assessed by interview and not with an objective assessment tool. Another limitation was that race/ethnicity interaction with nutrition program participation could not be assessed due to limited power. Despite these limitations, this study has several strengths: (1) the use of data from a national representative sample of US adults, (2) filling a gap in the literature by comparison of chronic diseases and functional limitations by participating versus not participating in nutrition programs, and (3) comparison of sociodemographics of participants and nonparticipants in a relatively large sample of older adults.

## 5. Conclusion

Older adults receiving congregate meals and/or government home-delivered meals had overall poorer physical function and health as compared to their counterparts who did not participate in nutritional services. Future studies should assess nutritional adequacy for older adults who participate in nutritional programs comparing sex and race/ethnicity. Clinical studies are needed to determine nutritional needs of older adults with obesity [24]. It is recommended that OAA meal programs provide adequate nutrition education and counseling that address and help to reduce food insecurity [24]. OAA meal programs should provide nutritionally dense meals and meal enhancements for older adults with undernutrition and possibly for obese, older adults with malnutrition [24, 29].

## Figures and Tables

**Table 1:** Demographics and health issues of the combined sample receiving/not receiving OAA meals.

Variable	Parameter	Percent (95% CI)
Received funded meals	Yes	10.1 (8.0, 12.7)
	No	89.9 (87.3, 92.0)

Sex	Male	44.0 (42.2, 45.9)
	Female	56.0 (54.1, 57.8)

Race/ethnicity	Mexican American	3.4 (2.1, 5.6)
	Other Hispanics	3.8 (2.6, 5.6)
	Non-Hispanic Blacks	8.6 (6.6, 11.3)
	Non-Hispanic Asians	4.1 (3.1, 5.5)
	Non-Hispanic Whites	80.0 (75.3, 83.6)

Marital status	Married/partnered	60.7 (57.6, 63.7)
	Single, widowed, or divorced	39.3 (36.3, 42.4)

Smoking status	Current smoker	8.3 (7.1, 9.7)
	Not smoking	91.7 (90.3, 93.9)

Body mass index category	Underweight/normal weight	29.0 (26.4, 31.7)
	Overweight	36.5 (34.4, 39.7)
	Mild obesity	21.2 (18.7, 23.9)
	Moderate obesity	9.1 (7.8, 10.5)
	High obesity	4.3 (3.2, 5.5)

Fear of running out of food	Yes	10.2 (8.9, 11.8)
	No	89.8 (88.2, 91.1)

Poverty	Yes	11.1 (9.4, 13.2)
	No	88.9 (86.8, 90.6)

Self-rated health	Poor to fair	23.2 (20.8, 25.9)
	Good to excellent	76.8 (74.1, 79.2)

Stroke	Yes	9.0 (8.1, 10.0)
	No	91.0 (90.0, 91.9)

Emphysema	Yes	5.3 (4.3, 6.6)
	No	94.7 (93.4, 95.7)

Memory problems	Yes	13.0 (11.5, 14.5)
	No	87.0 (85.5, 88.5)

Need equipment for walking	Yes	19.6 (17.4, 22.1)
	No	80.4 (77.9, 82.6)

Limited in amount of work can do	Yes	31.2 (27.9, 34.8)
	No	68.8 (65.2, 72.1)

*Notes*. Data presented as percent (95% confidence intervals). Parameters were *p* < .001 from each other for all variables, based on the adjusted *F*, a variant of the second-order Rao-Scott adjusted chi-square statistic and its degrees of freedom. Poverty was considered at <1.00 poverty index. Complex analysis for the population frequencies was based on the unweighted sample, *n* = 2,392. Significance was considered at *p* < .05. OAA: Older Americans Act.

**Table 2:** Comparison between participants who received and did not receive meals funded by the Older Americans Act (OAA).

Variable	Received meals funded by the OAA		*p*
	Yes	No	
Sex			
Male	33.4 (27.0, 40.5)	45.2 (43.6, 46.9)	.001
Female	66.6 (59.5, 73.0)	54.8 (53.2, 56.4)	

Race/ethnicity			
Mexican Americans	4.0 (1.8, 8.7)	3.4 (2.0, 5.0)	.105
Other Hispanics	2.9 (1.6, 5.1)	4.0 (2.6, 5.9)	
Non-Hispanic Blacks	12.7 (7.7, 10.2)	8.2 (6.2, 10.7)	
Non-Hispanic Asians	2.6 (1.4, 4.8)	4.3 (3.2, 5.7)	
Non-Hispanic Whites	77.7 (67.6, 85.4)	80.2 (75.2, 83.7)	

Marital status			
Married/partnered	38.8 (31.4, 46.8)	63.2 (60.2, 66.1)	<.001
Single, widowed, or divorced	61.3 (53.2, 68.6)	36.8 (33.9, 39.8)	

Smoking status			
Current smoker	7.6 (5.5, 10.5)	8.4 (7.0, 9.9)	.611
Not smoking	92.4 (89.5, 93.5)	91.6 (90.1, 93.0)	

BMI category			
Underweight/normal weight	32.0 (26.4, 38.3)	28.7 (26.1, 31.4)	.281
Overweight	36.6 (34.0, 43.4)	36.3 (33.9, 38.8)	
Obesity I	19.8 (15.2, 25.5)	21.4 (18.8, 24.2)	
Obesity II	6.5 (4.2, 9.9)	9.3 (8.0, 10.9)	
Obesity	3.1 (1.6, 6.0)	4.3 (3.3, 5.6)	

Fear of running out of food			
Yes	14.9 (11.8, 18.5)	9.7 (8.3, 11.4)	.002
No	85.1 (81.5, 88.2)	90.3 (88.6, 91.7)	

Poverty			
Yes	23.4 (17.7, 30.4)	9.7 (8.0, 11.9)	<.001
No	76.6 (69.6, 82.3)	90.3 (88.1, 92.0)	

Self-rated health			
Good to excellent	44.0 (38.6, 49.6)	29.8 (26.1, 33.7)	<.001
Fair to poor	56.0 (50.4, 61.4)	70.2 (66.3, 73.9)	

Stroke			
Yes	15.8 (11.1, 22.2)	8.3 (7.4, 9.2)	.001
No	84.2 (77.9, 88.9)	91.7 (90.8, 92.6)	

Emphysema			
Yes	9.4 (6.4, 13.6)	4.9 (3.9, 6.2)	.005
No	90.6 (86.4, 93.6)	95.1 (93.8, 96.1)	

Memory problems			
Yes	24.8 (20.4, 30.0)	11.6 (10.3, 13.2)	<.001
No	75.1 (17.0, 79.6)	88.4 (86.8 89.8)	

Need equipment for walking			
Yes	32.7 (27.7, 38.0)	18.1 (15.9, 20.6)	<.001
No	67.3 (62.0, 72.3)	81.9 (79.4, 84.1)	

Limited in amount of work can do			
Yes	44.0 (38.6, 49.6)	29.8 (26.1, 33.7)	<.001
No	56.0 (50.4, 61.4)	70.2 (66.3, 73.9)	

Data are presented as percent (95% confidence intervals). The percentages are weighted (sample weights, applied). Variables are totaled to 100% for “yes” and 100% for “no” responses. BMI: body mass index (kg/m^2^). Categories are defined as underweight (<18.5) to normal weight (18.5–24.9), overweight (25–29.9), Obesity I (30–34.9), Obesity II (35–39.9), and Obesity III (≥40). Poverty was considered at <1.00 poverty index. Significance was based on the adjusted *F*, a variant of the second-order Rao-Scott adjusted chi-square statistic and its degrees of freedom. Significance was considered at *p* < .05.

**Table 3:** Logistic regression analysis of demographics and health indicators for participation in OAA nutrition program compared to nonparticipation.

Variable	Parameter	OR (95% CI)	*p*
Gender	Male	0.83 (0.62, 1.11)	.203
	Female (reference)	1.00	—

Age (years)		2.23 (2.08, 1.17)	<.001

Poverty level	Index < 1.0	2.09 (1.18, 3.72)	.013
	(reference)	1.00	

Race/ethnicity	—	—	.245
	Mexican American	0.96 (0.44, 2.10)	.911
	Other Hispanics	0.52 (0.22, 1.23)	.131
	Non-Hispanic Blacks	1.34 (0.71, 2.54)	.359
	Non-Hispanic Asians	0.61 (0.26, 1.43)	.247
	Non-Hispanic Whites (reference)	1.00	—

Marital status	Married/partnered	0.52 (0.32, 0.86)	.013
	Other (single, widowed, or divorced; reference)	1.00	—

Current smoker	Yes	0.93 (0.59, 1.45)	.733
	No (reference)	1.00	—

BMI category	—	—	.506
	<25 (under/normal wt.)	1.50 (0.70, 3.20)	.286
	25–29.9 (overweight)	1.55 (0.72, 3.34)	.249
	30–34.5 (Obesity I)	1.39 (0.64, 2.98)	.391
	35–39.9 (Obesity II)	1.02 (0.43, 2.46)	.957
	≥40 (Obesity III, reference)	1.00	—

Food insecurity (fear of running out of food)	Yes	1.12 (0.75, 1.69)	.559
	No (reference)	1.00	

Self-rated health	Good to excellent	0.52 (0.36, 0.77)	.002
	Fair to poor (reference)	1.00	—

Stroke	Yes	1.33 (0.84, 2.10)	.213
	No (reference)	1.00	—

Emphysema	Yes	2.02 (1.05, 3.89)	.035
	No (reference)	1.00	—

Memory problems	Yes	1.25 (0.96, 1.64)	.095
	No (reference)	1.00	

Need equipment walking	Yes	0.97 (0.71, 1.32)	.830
	No (reference)	1.00	—

Limited in amount of work can do	Yes	0.92 (0.65, 1.30)	.634
	No (reference)	1.00	—

*Note.* Dependent variable: participation in congregate meals and/or government delivered meals. Nutrition program = home-delivered meals, congregate meals, or both. BMI: body mass index; wt.: body weight; OAA: Older Americans Act. Model fit was considered at *p* < .01 with correct classification of cases ≥ 75%.
